# Editorial: The psychology of tourism entrepreneurs

**DOI:** 10.3389/fpsyg.2022.1119778

**Published:** 2023-01-05

**Authors:** Rob Hallak, Craig Lee, Mike Mustafa

**Affiliations:** ^1^UniSA Business, University of South Australia, Adelaide, SA, Australia; ^2^Department of Tourism, University of Otago, Dunedin, New Zealand; ^3^Nottingham University Business School Malaysia, University of Nottingham, Selangor, Malaysia

**Keywords:** tourism, entrepreneur, COVID-19, psychology, industry

The COVID-19 pandemic has dealt a major economic blow to many small and medium-sized enterprises (SMEs) in the tourism sector (Dvouletý et al., 2021). The UNWTO estimated a loss of US $1.3 trillion in tourism revenues, as well as 100-200 million direct tourism jobs at risk due to the pandemic. Many of these SMEs in the tourism industry are predominantly independently owned, founded, and driven by entrepreneurs (Hallak et al., 2012). As risk and uncertainty have become increasingly volatile during this period (Jalonen, 2012), many entrepreneurs operating in the tourism industry are increasingly finding themselves making decisions in difficult circumstances. According to Williams (2021), entrepreneurs are not passive actors in responding to such circumstance; instead, to survive and recover entrepreneurs often re-evaluate and become innovative by effectively utilizing their resources and array of competencies while learning to grapple with uncertainty. Therefore, innovative responses to the COVID-19 crisis are largely contingent on individual entrepreneurs' human and social capital as well as their individual cognitions and attitudes (Dakhli and De Clercq, 2004).

In this regard, a psychological lens has much to offer to enhance our understanding of how entrepreneurs in the tourism sector have responded to the COVID-19 crisis and developed recovery strategies (Gorgievski and Stephan, 2016). However, to date there remains a general shortfall of empirical studies that have adopted psychological frameworks to explore the decisions and behaviors of entrepreneurs in tourism sector specifically (Williams, 2021). This special issue responds to shortfalls in the literature by bringing together a collection of articles that focus on tourism entrepreneurs through psychological frameworks. The five articles in this special issue adopt different psychological theories ranging from social-cognitive perspectives, entrepreneurial learning strategies and ultimately values to enrich our understanding of tourism entrepreneurs and their responses to the COVID-19 crisis. Below we provide a summary of each of the five articles and their contribution to understanding the psychology of tourism entrepreneurs.

Based on sample of 644 small tourism business owners from Malaysia, Jalil et al. examine the impact of psychological capital on organizational resilience of such business owners. Their findings show that psychological capital plays an important role in enhancing coping strategies, and that coping plays a vital role for owners/managers in the rehabilitation of small businesses during or after a crisis, implying that the psychological capital of owners/managers is critical in building organizational resilience. Such findings broadly demonstrate the importance of concepts such as resilience and optimism among tourism entrepreneurs in coping with the effects of COVID-19 on the tourism industry.

In the second article, Xu et al. use Airbnb's crawler panel data such as listing information, customer reviews, and booking calendars from 348 hosts for 5 years to explore impact of start-up age on host performance growth in the sharing accommodation sector. Their findings show that the relationship between a host's start-up age and its growth is initially positive before an inflection point and then turns negative, representing a curvilinear relationship. Additionally, product supply was found to moderate this relationship. At the early stage, operating and cost pressures from product supply, and at the late stage, using product supply as a risk reduction strategy, weakens the influence of age on growth. Collectively, Xu et al.'s study highlights the significance of the entrepreneurial learning from experience for entrepreneurs.

Utilizing a mixed method participatory-action research approach, Ferreira et al. explored whether the extent to which farmers' intention to be involved in tourism micro entrepreneurial activities is influenced by their social capital. A key finding in their study was that only tourism micro entrepreneurial self-efficacy had a significant relationship with entrepreneurial intention. This led the researchers to conclude that farmers' perceived efficacy in understanding the industry's regulation or getting adequate liability coverage does not significantly affect their intention to engage in farm tourism enterprise. Such a finding supports earlier evidence regarding the importance of individual cognitions in determining one's intentions to engage in entrepreneurial activities.

The fourth article by Quan et al. seeks to understand the fundamental perceptions of Chinese travelers' intentions of using vaccine passports. They find that the perceived usefulness of vaccine passports by travelers has significant positive effects on their attitudes toward travel abroad and attitude toward domestic travel. Although not examining tourism entrepreneurs directly, their findings have important implications on how tourism entrepreneurs can begin the recovery process during COVID-19. Specifically, the findings suggest the importance of individual entrepreneurs' ability to continuously scan their environment for opportunities and to provide innovative solutions that can gain consumers' trust in the face of uncertainty is vital to crisis recovery.

The final article in this special issue by Khan et al. takes an interesting approach to understanding how tourism entrepreneurs can potentially deal with the loss of employees during the COVID-19 pandemic. Based on data from 842 front-line employees in Pakistan, Khan et al. explore the relationship between Organizational Hypocrisy and Job Embeddedness and whether Perceived Inducement Breach and Future Anxiety mediate this relationship. They find that while organizational hypocrisy increases job embeddedness, it has negative psychological and emotional consequences through increasing Perceived Inducement Breach and Future Anxiety. Khan et al.'s study highlights the importance of tourism entrepreneurs in ensuring that their own individual values align with that of their employees through not only their words but also their actions. A miss-alignment can potentially lead to tourism entrepreneurs losing one of their most important resources and source of competitive advantage; their employees.

## Author contributions

All authors listed have made a substantial, direct, and intellectual contribution to the work and approved it for publication.
